# New Life Transition of Former Caregivers: Positive Mental Health Approach

**DOI:** 10.3389/fpsyg.2022.854108

**Published:** 2022-04-04

**Authors:** Gerard Mora-Lopez, Marta Berenguer-Poblet, Carme Berbis-Morelló, Nuria Albacar-Rioboo, Pilar Montesó-Curto, María Jesús Aguaron-García, Carme Ferré-Grau

**Affiliations:** ^1^Department of Nursing, Faculty of Nursing, University of Rovira i Virgili, Tarragona, Spain; ^2^Hospital Universitari Joan XXIII de Tarragona, Tarragona, Spain; ^3^Institut Català de la Salut, Atenció Primària Terres de l’Ebre, Tortosa, Spain

**Keywords:** caregiver, positive mental health, end of life, family care, qualitative study

## Abstract

After the end of their time as a caregiver, former caregivers have needs and feelings that have been subject to little study to date. The aim of the study is to determine and analyse the feelings, perceptions and practices of former caregivers in the reconstruction of their daily lives. This is a qualitative study based on the Grounded Theory developed by Charmaz. The study involved 14 former caregivers who had cared for their relative for more than 2 years and who had stopped caring for them more than 2 years previously. Fourteen in-depth interviews were conducted and data were collected over 13 months between 2015 and 2017. Data were analysed using the Grounded Theory Method. In addition, this study was approved by the ethics committee of the Institut Universitari d’Investigació en Atenció Primària Jordi Gol. The former caregiver experiences a transition, which begins in the days before the death of their relative and may continue for more than 3 years. Three critical moments in the post-caring transition were found: (1) the post-caring emptiness; (2) the end of the period as a caregiver; and (3) the movement towards a new life. Family and professional support is needed during this transition. Former caregivers experience a transition in the rebuilding of their daily lives; furthermore, former caregivers may be a source of support for other caregivers, which is linked to positive mental health factors. Healthcare organisations need to acknowledge the emotional, psychosocial and psychological health of former caregivers.

## Introduction

The increase in the aging of the population and improvements in health care have led to an increase in the proportion of the population with chronic diseases and dependent people. In Europe, chronic diseases cause 86% of deaths and affect 40% of the population over the age of 15 (Busse et al., 2010). Also, 88% of the care for dependent individuals is provided *via* an informal system of care, from a close relative, with no specific training for the care they provide, no financial remuneration and requiring high levels of dedication, 24 h a day, 365 days a year (Martínez-Marcos and De la Cuesta-Benjumea, 2014).

A close relationship is established between the caregiver and the relative, which gradually becomes a total commitment in which the caregiver gives up their opportunities to engage “almost exclusively” in caring for their relative. The caregiver gradually becomes immersed in the life and routines of the cared-for relative and a strong existential bond is established between the caregiver and the cared-for relative (Pereira and Rebelo Botelho, 2011).

Studies of family caregivers of chronically ill and/or dependent individuals have increased in recent years, particularly in the field of primary and/or home care. However, there is little evidence about the experience of former caregivers regarding the reconstruction of their daily lives.

This article attempts to answer the following research questions: What happens when a family caregiver stops providing care? What feelings, perceptions and needs do they experience? How do they (re)build their everyday life? This is the first study in our sociocultural milieu on the transition process among former caregivers.

Most publications study grief among former caregivers (Juozapavicius, 2001; Robinson-Whelen et al., 2001; Crespo et al., 2013) and describe two opposing models. Some authors, such as McGartland et al. (2001), advocate the model of relief after the death of the cared for relative and argue that after the person cared for has died, the caregiver is relieved of the burden of care and therefore their well-being improves. In contrast to this model, authors such as Ling et al. (2013) argue that post-caregivers show increased levels of depression 1 year after the death of their relative, due to the great existential void and emptiness that they experience.

Two of the qualitative studies describe the experiences and feelings of former family caregivers after the death of their relative. Larkin (2009) described three interrelated phases that former caregivers go through: (1) the post-caregiving emptiness phase, in which the former caregiver has to deal with changes in their life and particularly in their day-to-day routine, to cope with the pain or emptiness that the end of their time as a caregiver entails; (2) the phase of closure of the caring period, which involves the caregiver making a critical assessment of their caregiving experience and accepting it as a positive life experience; and (3) the phase of reconstructing life as a former caregiver, in which the former caregiver sees the light and tries to rebuild his or her daily life. These phases involve complicated and difficult transitions, which require the former caregiver to make an effective and continuous effort to adapt to everyday life.

Cronin et al. (2015) define three worlds: the pre-caring world, the caring world and the post-caring world. The study focuses on the feelings and needs of former caregivers in the transition from the caring world to the post-caregiving world. Three stages are identified from this transition, which coincide with those described by Larkin (2009). The loss of the world of caring is the first stage, in which the caregiver experiences many losses: the loss of identity as a caregiver, the loss of the person they care for and the loss of the social support network they had. The second phase, called living with loss, occurs when former caregivers report feelings of guilt due to the sensation of relief; in some cases, they are angry with their social and support network, as they feel abandoned. Many barriers to progressing towards the final stage emerge, such as financial problems and some caregivers will become trapped in the intrusive thoughts described in the Larkin (2009) model. Finally, the third phase (moving on) is when caregivers begin to move towards the new world. They begin to take care of themselves, become active, participate in community activities and get out of the home.

Recently, Corey and McCurry (2018) suggested that there may be long-term effects of caregiving on health that persist well beyond the first year post-caregiving. The study concludes that former caregivers would benefit from further research into the physical and psychological health of former caregivers after the first year post-caregiving. Moreover, Armstrong et al. (2019), who conducted telephone interviews with caregivers and family members of individuals who died with Dementia with Lewy Bodies, found a lack of communication between health care teams and families and discussed how it can affect the end of life process.

This transition has an impact on people’s mental health. Lluch (1999) proposed a multifactor model of positive mental health (PMH), comprising six factors that together constitute the PMH construct, which are: personal satisfaction, prosocial attitude, self-control, autonomy, problem solving and self-actualisation and interpersonal relationship skills.

PMH is defined globally as “a dynamic and fluctuating state in which the person tries to feel and be as well as possible within the circumstances in which he finds himself” (Lluch, 2008).

## Aim

To examine and describe the experience, feelings and strategies used by former caregivers on the process of reconstructing daily life after the grieving process.

## Materials and Methods

This study follows the constructivist grounded theory approach (Charmaz, 2006). Unlike the classical grounded theory method, this approach accounts for the context of the research itself and the position that the researchers have in it. It considers multiple social realities and recognises that knowledge is interpretative and constructed with the participants of the study. Data collection and analysis run simultaneously; data are explained through their interpretation and conceptualisation (Charmaz, 2009).

### Sample and Procedure

Fourteen former caregivers who had been family carers for at least 2 years and who had ceased to be a caregiver at least 2 years previously volunteered to take part in the research and were fully informed of all aspects of the study. The inclusion criteria were: (1) having cared for a dependent relative for more than 2 years; (2) being at least 18 years old; (3) having given informed consent to participate in the study; and (4) having stopped being a caregiver at least 2 years previously. The final criterion was established to capture the experiences of the different stages which former caregivers go through without interfering with the grieving process. Most of the participants were wives and parents with a high level of education. Their ages ranged from 40 to 87 and their relatives had been dead for between 3 and 20 years (see Table 1). The participants were contacted for the interviews with the help of the health professionals working in the health centres and in addition to the help of caregiver associations.

**TABLE 1:** Characteristics of the informants.

Code	Gender	Age	Marital status	No. Children	Education	Employment status	Person cared for	Main pathology	Years as a caregiver	Years since care ended Caregiver
CG1	Female	74	Widow	3	Primary	Retired	Husband	Cancer	5	3
CG2	Female	76	Widow	2	Primary	Retired	Husband	Cancer	7	3
CG3	Female	83	Widow	4	Illiterate	Retired	Husband	Parkinson’s	7	3
CG4	Female	55	Divorced	1	Secondary	Employed	Father/mother	Schizophrenia/Alzheimer’s	20	3
CG5	Male	71	Divorced	2	University	Retired	Mother	Alzheimer’s	13	15
CG6	Male	87	Widower	2	University	Retired	Woman	Traffic accident	30	2
CG7	Male	40	Single	0	University	Employed	Mother	Alzheimer’s	5	2
CG8	Female	62	Married	1	University	Employed	Daughter	Cerebral palsy	14	5
CG9	Female	76	Single	0	University	Retired	Mother	Alzheimer’s	20	4
CG10	Female	64	Single	1	University	Employed	Mother/father	P. Mobility	2	9
CG11	Female	66	Widow	3	University	Retired	Husband	Cancer	7	5
CG12	Female	72	Widow	2	University	Retired	Husband	Cancer	3	2
CG13	Male	53	Married	1	Secondary	Unemployed	Son	Rare disease	11	2
CG14	Male	53	Married	1	University	Employed	Son	Encephalopathy	9	5

*Compiled by the author.*

As the study developed, theoretical sampling took place to reach the saturation of categories (Strauss and Corbin, 1998). Thus, former caregivers with different family relationships, educational backgrounds and relatives they had cared for were searched and the interview guide was modified to saturate emerging categories.

### Data Collection

A total of 14 semi-structured interviews (Kvale and Brinkmann, 2009) were conducted between May 2017 and June 2018. Interviews lasted between 50 and 90 min and were recorded and transcribed in full. Interviews began with exploratory questions that became more specific as the codes and categories were developed. A script was prepared based on the literature review and modified during the research process (see Box 1). Emphasis was placed on the family former caregivers’ everyday life and their experiences and feelings between the death of their relative until the day of the interview. At the end of the interview, the researcher repeated the core question, giving the informant the opportunity to reflect upon and further expand their description. Data collection ended with the saturation of categories.

**Box 1. Guide for interview.**

Caregiver period

• What did caring for your relative mean?
• Process of dying
• How did you experience the time when you stopped providing care?
• What were the main changes in your life when you stopped providing care?

Financial situation

• General situation
• Work Assess return to work
• Social condition

Health

• Health problems (physical/mental)
• How you felt after the caregiving period
• Did you feel that your health improved after you stopped providing care? How?
• Changes in health
• Use of services

Family and social life

• Did your relationship with your relatives change after you stopped providing care?
• Changes in daily routine
• How do you experience traditional events? (Christmas, birthdays, family events)
• Networks of caregivers
• Do you have any hobby or interest since caring for your relative?
• How do you spend your time?
• How do you think you would help people in the same situation as you?

Emotions

• Feelings experienced (Questions such as: How do you feel now? Different feelings during the process)
• What helped you during the transition?
• Beliefs
• Roles
• Helping other caregivers

### Data Analysis

The analysis was conducted as the data were collected. The analysis and coding process was as follows:

a) Collection of empirical data. The appropriate techniques for approaching the informants through our own sources of primary care nurses as well as various associations of patients and relatives were used in this first step in the application of the method. Transcription of the interviews began at the same time as the data collection process. Line-by-line analysis was performed using the constant comparison method.
b) Codification. This is the procedure by which the data collected were reordered, related and conceptualised by the researchers. During the process, the data began to be sorted, conferring the necessary methodological rigour on the scientific process, identifying nuances and developing the foundation, density, sensitivity and integration necessary to generate the theory.

According to grounded theory, data analysis is a dynamic and creative process (Glaser and Strauss, 1967; Dantas et al., 2009). At the end of the analysis, we obtain a deeper understanding of the issue of the transition of post-care and the reconstruction of the daily life of former caregivers.

The use of textual references and their conceptualisation, as well as the clear description given of the different stages of the research, contribute to the credibility of the results and their transferability. The data and results underwent a process of analysis to ensure the validity of the study. The authors used an audit trail. First, there were contributions by a multidisciplinary group of experts composed of nurses, psychologists and social workers for the various associations participating in the study. Second, we performed a requested validation, i.e., those involved had access to the lead researcher’s interpretation based on the data generated and were able to corroborate the reading of the context and the interpretation. Furthermore, all of the researchers read and analysed the data independently.

### Ethical Consideration

Ethical approval was obtained in June 2014 from the Healthcare Ethics Committee of the Jordi Gol Primary Healthcare Research University Institute in Barcelona (Spain). To preserve anonymity, pseudonyms are used in the text below. All of the names and places that appear in the text are fictitious. In addition, informed consent was obtained from the participants.

## Results

In the data analysis, it was identified that former caregivers experience a transition which begins in the days before the death of their relative and can continue for more than 3 years. Three critical moments in the transition from former carer were found: (1) the emptiness in post-caring; (2) the end of the period as a caregiver; and (3) the movement towards a new life (Table 2). An analysis of the content of each category is presented below.

**TABLE 2:** Relationship established between categories and PMH factors (Lluch, 1999).

Category	Concepts	PMH factor	Verbatim
Emptiness of post-caring	Emotional emptiness Instrumental emptiness	F1: Personal satisfaction F3: Self control	*F1: “Thousands of messages on Facebook, acquaintances and greetings., and I don’t remember ever having embraced so many people or having so many people embrace me as on the day of his funeral. And I think we feel this energy” (CG14)* *F3: “I always feel lonely, but I think that people are born and live alone, you have to adapt to a variety of circumstances, but we always live alone” (CG6).*
End of the period as a caregiver	Routines of closure. Post-caregivers’ health. Support received during closure.	F2: Prosocial attitude F3: Self-control	*F2: “Sometimes I went to see Pedro because I felt sorry for him and he was pleased to see me but the poor man was in a very bad way”*
Movement towardss the new life	Recovery of activities Joining the labour market. Feeling good about oneself.	F5: Problem-Solving and Self-Actualisation F4: Autonomy F6: Interpersonal Relationship Skills	*F4: “The second year was fine. Besides, I’ve always travelled a lot and I carried on travelling.” (CG11)*

*Complied by the author.*

### The Emptiness in Post-caring

The concept of emptiness emerged spontaneously in all interviews. They described it as an indescribable existential phenomenon, with former caregivers mentioning a *lack of meaning in their life*, while others compared it to being submerged in a *black hole.* All informants highlighted the concept of loss.

*“Somebody’s death is an emptiness. A very large emptiness. I would call it an existential emptiness”* (CG8).

In the study population, the concept of emptiness consists of an instrumental factor, which refers to the lack of the aspects related to care tasks and routines, as well as physical space and an emotional factor determined by the end of the bond between the caregiver and their relative. The former caregiver must break the strong connection that has been established with the dependent person and start their new life, beginning once again to establish goals, routines and ambitions that they have neglected for a long period of time. The first feeling they reported related to emptiness was loneliness:

*“I always feel lonely, but I think that people are born and live alone, you have to adapt to a variety of circumstances, but we always live alone*” (CG6).

Loneliness increases and prolongs in time the feeling of emptiness as a result former caregivers need the company of their family.

During this transitional phase, the post-caregiver must find a new purpose in their life, achieve new goals and giving meaning to new routines and activities that will allow them to restore their spirits and fill the void on a physical, social and emotional level.

There is also a loss of control over time management, since their life during the caregiving period was structured around the care of the relative, i.e., their time was determined by various tasks related to hygiene, food, support and basic care for the relative; subsequently their time organisation has been completely broken down:

“*You find an enormous emptiness, you’ve been caring for someone for 24 h a day for 5 years and you suddenly find that you no longer have that responsibility, so you’re on your own with your life and with all the time in the world to decide what to do with it*” (CG7).

Although the grieving process is part of the emptiness in post-caring, this feeling of emptiness lasts for some time, while grief is overcome within approximately a year and a half (Tizón, 2013). In addition to the process of mourning the loss of the family member, the emptiness in the task of post-caring is identified as broad-based throughout the transition process and has a positive or inhibiting effect.

Post-caring emptiness sets different factors of PMH in motion. First, the self-concept is affected, which is included in the personal satisfaction factor. The void generates a loss of meaning in life since there is a step towards a new life and the person must break with the life as a caregiver. In this way, self-control mechanisms are activated and the post-caregiver has to cope with stressful situations.

### End of the Period as a Caregiver

This category includes the activities or processes for closure that emerged from the discourse of the former caregivers. The first time that most of them became aware of the end of their time as a caregiver was on the day of the funeral. The former caregivers retained an emotional memory of the ceremony and experience it as a tribute to their relative and their work.

“*Thousands of messages on Facebook, acquaintances and greetings. and I don’t remember ever having embraced so many people or having so many people embrace me as on the day of his funeral. I think we feel this energy*” (CG14).

The closure stage contains routines and tasks directly related to the death, such as administrative, financial and legal formalities, as well as getting rid of the belongings of the deceased person that are no longer needed. Although this ritual is part of the routines for closure when anyone dies, in the case of caring for the dependent population, this stage is increased by the volume of technical aids and medical equipment used during the caring. All of these materials are redistributed to individuals and institutions that need them, although post-caregivers have very strong memories of the difficulties involved in obtaining them in the past as well as their high financial cost. All of the interviewees agree that this emptiness appears within 3 months.

“We *gave all the technical aids to hospital*” (GC5).

There is a wide variety of administrative and governmental institutions, with all of the participants mentioning difficulties in carrying out administrative procedures after their relative’s death, the slowness and complexity of the administrative procedures, which led to strong feelings of powerlessness and anger, as well as sadness and anxiety about the death of their relative which enhanced their unhappiness. These results highlight the need to improve policies to be able to make post-mortem formalities more flexible.

Another phenomenon that emerged was “*staying connected during the closure*”; i.e., in this stage of closure, former caregivers sought something to keep them connected in some way with their past life as a caregiver to fill the emptiness and remember their work. As Ángeles explains, her husband was admitted to hospital; when he died, she went to visit him and took care of his roommate:

“*Sometimes I went to see Pedro because I felt sorry for him and he was pleased to see me but the poor man was in a very bad way* [.] *he was my husband’s roommate and I was very sorry for him, a young boy and with that life and it was no skin off my nose and he was glad to see me and that way I saw people at the hospital*” (CG3).

Nine of the fourteen participants repeated similar behaviour. While it did not take place in the hospital, they sought neighbours or relatives to help and offer their support to as expert caregivers in order to keep a link to caring.

It is this phase the pro-social attitude is activated. Former caregivers take on the role of helping other caregivers, becoming a source of support and increasing personal satisfaction. Due to the changes experienced in this phase, defined by some authors as a crash, health problems may appear, including depressive syndromes like those described by Hash (2006) and Kim (2009). Four caregivers in our sample implicitly said that they suffered from stress and depression. Depression in former caregivers consists of biopsychosocial changes related to age and loss (Montesó et al., 2012):

“*I started to have a lot of somatic symptoms, in terms of illness, stress levels, gallstones and not eating; I even got depression*” (CG10).

Regarding the support perceived by the informants in our socio-cultural context, all of them reported having good support from family and friends, which is in contrast to the results of Larkin (2009) and Cronin et al. (2015). However, a lack of professional support was apparent, meaning that no specific support from health professionals is perceived.

One of the most problematic issues in the transition for former caregivers was comments made by acquaintances about the well-deserved rest due to their time as a caregiver having ended.

*“An obsession that people have, who say ‘well, at least now you’ll be more relaxed, you won’t have so much work.’ And you thought: ‘Don’t tell me that.”’* (CG14).

The participants reported a complete misconstruction of reality, or completely opposite points of view. On the one hand, there are people who have never been caregivers or whose experience is far away from the daily life of caregivers, who often see the world of caring for a disabled person throughout their entire life as a difficult process with a high emotional cost. On the other, there are former caregivers who are immersed in the process of breaking up and reconstructing their lives, where their life’s purpose (to take care of their dependent child) has come to an end. Consequently, what society considers as a relief is experienced by former caregivers as a misfortune; this contradictory perspective may lead to moral confrontations that affect their relationships with other people. These discrepancies require flexibility to adapt to change. An attitude of continuous growth and personal development is important; problem solving and self-realisation are observed in this phase.

### The Movement Towards a New Life

This concept refers to how former caregivers begin to mobilise their energies to rebuild a new life. The strategies that helped participants in the transitions include activities related to self-care, staying active, starting to become involved in social activities and becoming aware of changing situations. These results have similarities with those of Cronin et al. (2015). The phases of the transition are non-linear, so each person experiences their own process and begins this movement at their own pace. The discourse includes indications that refer to the tempo each person needs to resume activities, travel and recover friendships, among other activities. It is a complex transition, in which former caregivers begin to move towards the reconstruction of their new daily lives. The informants defined their transition in terms of time and movement as follows:

“*The second year was fine. Besides, I’ve always travelled a lot and I carried on travelling*” (CG11).

An interesting fact is that more than a third of the participants mentioned a third year, in which there is a sense of improvement and when they start rebuilding their lives.

Some of the difficulties that former caregivers experience during this phase are changes in their friendships, as their social relations change during the caregiver period; once the period is over, they have to re-establish new ones. However, these will not be as close as in their period as a caregiver in most cases, as they shared very intimate aspects of care related to hygiene, elimination, feeding, difficulties in communicating etc., during the caring period with other family members and professionals.

During this period, the former caregiver also feels that they are going through a phase in which they have to redefine their personal projects, family relationships and relationships with the employment world. These issues are particularly important when the person has neglected their personal projects for many years in order to take care of their relative.

Former caregivers must leave behind the life of the family member they cared for and take charge of their own. People increasingly find ways to spend their time redefining their roles and social relationships, so the windows from the caregiver period close and a daily life defined by new routines and new relationships with others is (re)constructed.

Former caregivers have to break the link they had created with their relative and deal with the discrepancies that arise in the task of post-care, must go to work to occupy their time and must find a new purpose in life.

The last phase is where a new world is built, where former caregivers must understand their new life. At this stage, they must develop all PMH factors to facilitate their mental well-being.

## Discussion

Most of the studies included in the research literature provide substantial evidence of the effects of the death of the relative, but very few describe the needs, feelings and perceptions experienced by the family caregiver in the reconstruction of their life as a family former caregiver.

This study has described the transition from post-caring and the reconstruction of the everyday life of former caregivers in our sociocultural environment. The study was conducted in Spain with middle-aged participants who had cared for their relatives. The findings might be different in another culture and context. However, there are similarities with recent qualitative studies performed by Larkin (2009); Cronin et al. (2015) and Corey and McCurry (2018) in the English-speaking environment. Some of the commonalities between these studies and this paper relate to the transition phases.

One of the aspects in our findings that is consistent with previous authors (Larkin, 2009; Cronin et al., 2015) refers to the loss of the caregiver role, consisting of factors such as the end of tasks from caring and a loss of recognition and power within the family as a caregiver. Most participants define this as a loss of purpose in life. In Spain, the caregiver assumes full responsibility for caring and a strong bond is established between the caregiver and the family member cared for, where the caregiver experiences depersonalisation because they neglect their own life in order to devote themselves to caring for their relative (Pereira and Rebelo Botelho, 2011).

All of the studies agree on three main phases characterised by a psychological and instrumental emptiness, a phase of closure in the caregiver’s activities and life and a phase of moving towards a new life defined by the reconstruction of everyday life. There is a big difference in the time frame of the transition. Our findings regarding the timing are in contrast with those of Larkin (2006), who stated that the transition lasted for approximately 1 year; in the sample of this study, the transition lasted for over 3 years, perhaps because of the time spent caring for the relative and the strength of the relationship established. During these years, the former caregiver must overcome grief, find closure for their time as a caregiver and search for a new purpose in life.

One of the differences between this work and the study of Cronin et al. (2015) is the characteristics of the sample; the previous authors considered former caregivers to be people who stopped providing care when their relative was admitted to a nursing home. In our cultural context, this profile of former caregivers has not been included because families still have a strong presence in hospitals, as reported by previous studies (Mora-Lopez et al., 2016); we believe it is interesting to analyse this phenomenon to identify similarities and differences.

Another item of interest is the need to implement a training programme for healthcare professionals and design a specific programme for attention to former caregivers, as well as to evaluate its effectiveness.

Another point of interest is the need to implement a training programme for healthcare professionals and design a specific care programme for former carers, as well as evaluating its effectiveness.

At the end of the family caregiving period, family former caregivers experience a multifaceted transition in which they have to redefine their identity and rebuild their daily lives. This transition starts before the relative’s death and continues beyond the grieving process. During this process, former caregivers express multiple experiences and feelings that are crucial to the reconstruction of their new identity as former caregivers. The fact that caregivers have ongoing emotional needs post-care indicates that this stage should be considered as part of the caregiver’s life (Orzeck and Silverman, 2008).

The emptiness of the post-caring task takes shape in the transition from post-caring and involves multiple factors: an instrumental process related to the (re)organisation of time and the loss of meaningful tasks, routines and activities and the emptiness in the physical space. In addition, there is an emotional process related to the various losses: the death of the relative, the loss of their role and power in the family and all that forms an existential emptiness.

According to Corey and McCurry (2018) and Armstrong et al. (2019), there is an evident lack of medical and social support in the transition from family post-caring. Medical support should be led by nurses, as they are the closest figure to families and they are the ones who have established a strong bond during the caregiving period. This implies a multidisciplinary effort to get to know the person: assessing each process of transition to create an individual profile of the client’s readiness and thus empowering the individual to create the optimum conditions for the transition (Meleis et al., 2000). Finally, we believe that work on the family post-caregiver should continue, as well as research to create further evidence that can guide care for families and post-caregivers.

According to other authors, the post-caregiving period should be viewed as an integral part of the caregiving career, with recognition that former carers continue to have practical and psychological needs once caregiving comes to an end (Cavaye and Watts, 2016).

Transition from caregiver to post-caregiver presumes an instrumental and emotional transition in people’s lives. The Positive Mental Health model (Lluch-Canut et al., 2013; Lluch, 2015) can be used to understand some emotional changes as well as to establish a therapeutic plan aimed at promoting positive mental health in former caregivers (Table 2).

## Limitations

It is plausible that the transition of former caregivers may be different in other sociocultural situations. Another limitation is that the caregiver period ending when the relative enters a nursing home has not been taken into consideration, as caregivers have a very active presence in hospitals and nursing homes in Spain, unlike in other countries.

## Data Availability Statement

The datasets presented in this study can be found in online repositories. The names of the repository/repositories and accession number(s) can be found below: https://www.tesisenred.net/handle/10803/399228.

## Ethics Statement

The studies involving human participants were reviewed and approved by IDIAP JORDI GOL. The patients/participants provided their written informed consent to participate in this study.

## Author Contributions

All authors listed have made a substantial, direct, and intellectual contribution to the work, and approved it for publication.

## Conflict of Interest

The authors declare that the research was conducted in the absence of any commercial or financial relationships that could be construed as a potential conflict of interest. The handling editor declared a past collaboration with one of the authors CF-G.

## Publisher’s Note

All claims expressed in this article are solely those of the authors and do not necessarily represent those of their affiliated organizations, or those of the publisher, the editors and the reviewers. Any product that may be evaluated in this article, or claim that may be made by its manufacturer, is not guaranteed or endorsed by the publisher.
